# Parcellation‐based anatomic modeling of the default mode network

**DOI:** 10.1002/brb3.1976

**Published:** 2020-12-18

**Authors:** Zainab Sandhu, Onur Tanglay, Isabella M. Young, Robert G. Briggs, Michael Y. Bai, Micah L. Larsen, Andrew K. Conner, Vukshitha Dhanaraj, Yueh‐Hsin Lin, Jorge Hormovas, Rannulu Dineth Fonseka, Chad A. Glenn, Michael E. Sughrue

**Affiliations:** ^1^ Department of Neurosurgery University of Oklahoma Health Sciences Center Oklahoma City OK USA; ^2^ Centre for Minimally Invasive Neurosurgery Prince of Wales Private Hospital Sydney NSW Australia; ^3^ Cingulum Health Sydney NSW Australia

**Keywords:** ALE, anatomy, default mode network, parcellation, tractography, white matter

## Abstract

**Background:**

The default mode network (DMN) is an important mediator of passive states of mind. Multiple cortical areas, such as the anterior cingulate cortex, posterior cingulate cortex, and lateral parietal lobe, have been linked in this processing, though knowledge of network connectivity had limited tractographic specificity.

**Methods:**

Using resting‐state fMRI studies related to the DMN, we generated an activation likelihood estimation (ALE). We built a tractographical model of this network based on the cortical parcellation scheme previously published under the Human Connectome Project. DSI‐based fiber tractography was performed to determine the structural connections between cortical parcellations comprising the network.

**Results:**

Seventeen cortical regions were found to be part of the DMN: 10r, 31a, 31pd, 31pv, a24, d23ab, IP1, p32, POS1, POS2, RSC, PFm, PGi, PGs, s32, TPOJ3, and v23ab. These regions showed consistent interconnections between adjacent parcellations, and the cingulum was found to connect the anterior and posterior cingulate clusters within the network.

**Conclusions:**

We present a preliminary anatomic model of the default mode network. Further studies may refine this model with the ultimate goal of clinical application.

## Graphical abstract

Using attentional task‐based fMRI studies to generate an activation likelihood estimation (ALE), we built a tractographical model of this network. Seventeen cortical regions were found to be part of the DMN: 10r, 31a, 31pd, 31pv, a24, d23ab, IP1, p32, POS1, POS2, RSC, PFm, PGi, PGs, s32, TPOJ3, and v23ab. These regions showed consistent interconnections between adjacent parcellations, and the cingulum was found to connect the anterior and posterior cingulate clusters within the network.

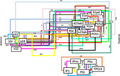

## 1 INTRODUCTION

Advances in human neuroimaging techniques have elucidated complex neural networks which execute key functions (Beckmann et al., 2005; Cole et al., 2014; De Luca et al., 2006; Razlighi, 2018; Smith et al., 2013; Thirion et al., 2006). A collection of interacting networks has been described in the literature, consisting of the brain's default mode network, salience network, and executive control network. In contrast to primary cortical areas which can be preserved during brain surgery, preservation of higher cognitive networks has proven more difficult due to their complex anatomy (Burks et al., 2018). Improvements in the understanding of the connectivity and anatomy of higher‐order cerebral networks are therefore likely to manifest in advances in brain tumor surgery.

Several studies have characterized the anatomy of the default mode network (DMN) since its discovery in 2001 (Andrews‐Hanna et al., 2010; Buckner et al., 2008; Horn et al., 2014; Raichle et al., 2001). It serves a primary role in passive states of mind, though it is also active during some goal‐oriented tasks (Bressler & Menon, 2010; Chand et al., 2017; Fox, Snyder, et al., 2005; Greicius et al., 2003). The network is typically described as consisting of the anterior and posterior cingulate cortices, and the lateral parietal lobe bilaterally (Alves et al., 2019; Andrews‐Hanna et al., 2010; Buckner et al., 2008). While important, existing descriptions of the DMN offer limited anatomical specificity, making it difficult to compare findings among different papers. This study instead relies on newly published parcellated brain maps to study the network anatomy of the DMN using a standard cortical atlas and nomenclature (Glasser et al., 2016).

In this study, a new cortical model of the DMN was constructed based on the parcellation scheme previously published under the Human Connectome Project (HCP) (Glasser et al., 2016). The HCP atlas is among the most detailed in vivo parcellation scheme constructed by combining automated machine learning approaches with extant neuroanatomical literature. It allows consistent and detailed delineation of cortical areas which we employed for its potential for reproducibility across studies and in clinical contexts. After identifying the cortical regions of interest involved in the network, we performed DSI‐based fiber tractography to demonstrate the structural connections between parcellations within the network. Our goal is to move toward a more precise anatomic model of the DMN for use in future studies.

## 2 METHODS AND MATERIALS

### 2.1 Literature search

We initially searched for relevant fMRI studies related to the DMN in BrainMap Sleuth 2.4 (Fox, Laird, et al., 2005; Fox & Lancaster, 2002; Laird et al., 2005). No research articles were identified using this software. Other literature software's were not queried as PubMed was subsequently queried to cover the literature gap on October 1, 2020, for fMRI studies relevant to the default mode network. We used the following search algorithm: (default mode OR default mode network OR DMN) AND “resting‐state fMRI” AND controls.” Studies relevant network were reviewed and included in our analysis if they fulfilled the following search criteria: (a) peer‐reviewed publication, (b) resting‐state fMRI study examining the DMN, (c) based on whole‐brain, voxel‐wise imaging, (d) including standardized coordinate‐based results in the Talairach or Montreal Neuroimaging Institute (MNI) coordinate space, and (e) including at least one healthy human control cohort. Only coordinates from healthy subjects were utilized in our analysis. Twenty‐eight papers met criteria for inclusion in this study (Anderson et al., 2011; Che et al., 2014; Chen et al., 2017; Chiong et al., 2013; Clemens et al., 2017; Crittenden et al., 2015; De Luca et al., 2006; Doll et al., 2015; Fransson, 2006; Greicius et al., 2003; Horn et al., 2014; Kennedy & Courchesne, 2008; Konishi et al., 2015; Laird et al., 2009; Lin et al., 2017; Luo et al., 2016; Maresh et al., 2014; Mason et al., 2007; Piccoli et al., 2015; Pletzer et al., 2015; Poerio et al., 2017; Spreng & Schacter, 2012; Stawarczyk et al., 2011; Taruffi et al., 2017; Utevsky et al., 2014; Vatansever et al., 2015; J. Xu et al., 2014; Yang et al., 2012). The details of these studies are summarized in Table 1.

**TABLE 1:** Studies used to generate the activation likelihood estimates of connectivity in the Default Mode Network

Study	Task	Number of participants	MNI/Talairach	Coordinates		
Anderson et al., 2011	Subjects were instructed, “Keep your eyes open and remain awake and try to let thoughts pass through your mind without focusing on any particular mental activity.”	57 (100%)	MNI	−4	−52	32
				4	−53	35
				−2	55	−13
				2	55	−13
				−49	−62	34
				50	−57	36
Che et al., 2014	Perceived social support at resting state.	333 (100%)	MNI	0	−45	36
				6	48	−18
				−51	−66	30
				48	−63	39
				−6	54	45
				6	45	−9
				−36	−69	42
				39	−66	42
				−6	−54	45
				−3	54	3
				−51	−69	33
				48	−66	45
Crittenden et al., 2015	Switch between similar and dissimilar tasks, within a relatively large set of six tasks.	18 (100%)	MNI	−30	−36	−6
				33	−36	−9
				−21	−42	9
				30	−39	6
				−9	−48	12
				9	−51	12
				−12	−54	24
				12	−51	24
				−9	51	−6
				9	48	−3
				−39	−75	33
Luo et al., 2016	To rest with eyes closed.	148 (100%)	MNI	0	51	39
				−6	−42	42
				−33	−60	54
Utevsky et al., 2014	Three reward‐based decision tasks requiring externally focused attention and resting state.	209 (100%)	MNI	15	−63	18
				0	−54	45
				−12	−69	18
				6	−42	3
				−3	−45	6
Vatansever et al., 2015	Finger opposition paradigm with task and fixation periods versus. independent resting state.	22 (100%)	MNI	0	52	26
				−6	52	−2
				0	26	−18
				−44	−74	32
				44	−74	32
				−54	−54	28
				54	−54	28
				−60	−24	−18
				60	−24	−18
				0	−58	27
				−14	−52	8
				14	−52	8
				−27	−15	26
				27	−15	26
				−28	−15	−12
				28	−15	−12
Doll et al., 2015	To remain still with eyes closed and to not fall asleep during acquisition.	26 (100%)	MNI	−6	53	1
				−6	47	−8
				6	47	7
				0	−52	28
				6	61	28
				−6	−52	28
				−6	−61	34
				12	−49	34
				12	−67	34
				9	−70	37
				−6	−76	34
Clemens et al., 2017	Participants believe that they play a virtual ball tossing game with two other participants to whom they are supposedly connected via a computer, resting‐state data taken before and after game.	89 (100%)	MNI	−6	28	32
				50	−14	26
				42	36	8
				22	8	50
				48	12	12
				4	−62	18
				18	−74	26
Pletzer et al., 2015	Four different tasks in a paper and pencil design to evaluate mathematical skills. Independent resting state.	36 (100%)	MNI	27	−42	−15
				−24	−45	−15
				57	−3	−15
				−60	−9	−15
				−51	−51	18
				−27	45	36
				33	42	30
				−18	66	3
				9	54	−6
				39	−15	−6
				−42	−57	21
				−18	−6	21
				−6	57	3
				54	33	3
				−9	−45	36

Lin et al., 2017	Visual‐based attention task and then a resting‐state scan.	14 (100%)	MNI	0	−53	26
				−3	55	22
				−3	59	−7
				−24	33	−27
				30	−33	−27
				−52	69	26
				48	−67	36
				−21	32	47
				12	44	48
				−61	−17	−30
				61	−5	−25
Taruffi et al., 2017	Listening to 4‐min blocks of sad and happy music (with the same tempo) with eyes closed.	24 (100%)	MNI	−6	39	−8
				15	39	−2
				0	45	31
				0	−36	52
				21	−51	16
				−48	−69	43
				54	−63	28
				48	12	25
Stawarczyk et al., 2011	Participants reported their conscious experiences in terms of both task‐relatedness and stimulus‐dependency while they performed the SART (Sustained Attention to Response Task). Independent resting state	22 (100%)	MNI	0	58	−2
				−8	−62	20
				−38	−80	36
				−16	−48	0
				48	38	36
				−60	−48	48
				2	58	−2
				−2	70	20
				−2	−50	22
				−44	−72	48
				−64	−22	−26
				52	−14	−32
				−64	−44	−8
				−52	26	24
De Luca et al., 2006	Resting state, instructed to relax with their eyes closed, without falling asleep	26 (100%)	Talairach	6	−78	−3
				24	−78	−10
				−30	−89	20
				−2	−51	27
				53	−57	23
				2	54	−3
				−20	−19	−18
				6	−19	6
				−4	−6	40
				−51	−7	8
				−55	−18	8
				57	−5	20
				12	−17	0
				22	−16	−13
				−2	−21	43
				46	6	34
				44	−48	46
				−38	−56	48
				−55	−58	−9
				62	−37	−3
				52	26	−4
				6	46	18
				8	−46	41
				8	−16	40
Greicius et al., 2003	Keep their eyes closed and to not think of anything in particular for 4 min.	14 (100%)	Talairach	−2	−51	27
				−51	−65	27
				−2	55	−18
				−16	49	38
				−44	20	41
				−12	−35	0
				18	54	32
				−58	−18	14
				2	38	−2
				2	−51	27
				4	−14	34
				4	9	−6
				4	−16	−3
				−4	−55	25
				0	−49	30
				−4	−49	26
Horn et al., 2014	Participants were asked to close their eyes.	19 (100%)	Talairach	6	−61	34
				−36	−63	47
				45	−43	52
				57	−28	25
				6	−58	34
				−54	−66	32
				45	−43	49
				60	−28	25
				9	−52	31
				−39	−46	43
				48	−46	28
				−3	62	−8
				−30	−100	4
				15	−64	32
				−36	−45	49
				39	−40	46
				0	68	0
				−30	50	7
				−31	−100	8
Maresh et al., 2014	Underwent two runs of the MID task, designed to assess one's neural response to the anticipation and receipt of rewarding or punishing monetary stimuli.	84 (100%)	Talairach	−18	−40	36
				−30	−56	36
				−8	−54	48
				0	−78	52
				−34	−56	42
				−6	−54	36
				−20	44	18
				−26	34	42
				−20	42	10
				−28	56	12
				−22	34	28
				−22	16	36
				−8	−56	48
				−10	−66	34
				−8	−42	48
				−6	−54	38
				−6	−50	34
				−10	−52	38
				−36	−42	54
				−52	−32	50
				8	−48	60
				−36	−54	54
				−42	−48	56
				−46	−50	56
				−50	−50	50
Fransson, 2006	Two 10‐min scans during which they performed a resting‐state, low‐level baseline task (eyes open, visual fixation on a hair‐cross‐centered in the screen) and a sequential two‐back verbal working memory task.	14 (100%)	Talairach	−39	−75	45
				−3	−63	48
				3	−66	30
				−33	18	57
				33	33	51
				−6	39	39
				9	45	−12
				−12	45	−12
				−60	−27	−15
				−57	−36	−6
				−27	−27	−24
				−42	9	−42
				63	−18	−21
				48	9	−42
				63	−27	−12
				39	−72	−42
				6	−60	−51
				−6	−57	−48
				30	−18	−24
				−24	−90	−27
				−27	27	−24
				57	9	12
				45	12	6
				66	−21	33
				63	−18	24
				69	−15	18
				−66	−27	18
				−36	3	9
Piccoli et al., 2015	Subjects were asked to judge whether a given target stimulus had been part of a previous memory stimulus set or not.	14 (100%)	Talairach	2	−44	31
				−2	25	−8
				41	−56	33
				−54	−63	20
				28	−56	47
				−25	−59	45
				44	3	24
				−55	5	30
				−2	−48	24
				2	46	9
				50	−59	23
				−44	−71	30
				35	−58	50
				−18	−70	46
				40	18	35
				−27	31	28
				−10	−53	13
				2	46	21
				46	−70	26
				−50	−65	38
				40	−53	48
				−33	−53	47
				43	22	27
				−38	16	24
				−4	−37	36
				−3	28	−3
				58	−53	24
				−55	−64	25
				33	−56	44
				−36	−38	37
				35	21	38
				−45	22	32
				2	−44	30
				−7	37	9
				59	−29	24
				−61	−38	15
				30	−53	30
				−40	−50	33
				32	43	28
				−55	16	33
				−3	−48	26
				−3	41	30
				30	−81	36
				−43	−74	32
				44	−32	36
				−36	−54	44
				42	38	39
				−41	30	38
				−1	−40	28
				1	29	−6
				41	−77	38
				−50	−74	20
				23	−67	48
				−34	−62	42
				35	29	25
				−21	35	28
				−3	−54	20
				4	52	11
				56	−58	32
				−53	−58	21
				24	−50	47
				−29	−50	41
				22	28	28
				−36	25	26
				0	−50	31
				−1	27	−13
				54	−63	24
				−46	−65	26
				17	−66	54
				−22	−64	51
				44	35	27
				−43	24	33
				−3	−42	29
				0	37	−3
				39	−69	28
				−52	−70	24
				30	−57	39
				−38	−44	36
				44	30	33
				−28	28	33
				−9	−51	26
				−2	31	−4
				53	−64	21
				−46	−75	24
				26	−66	36
				−37	−37	38
				34	19	36
				−31	19	26
				−10	−55	32
				−4	30	−5
				39	−71	34
				−48	−77	30
				31	−44	37
				−31	−50	33
				51	28	32
				−49	25	33
				−8	−35	33
				10	34	5
				50	−65	30
				−54	−65	28
				37	−74	35
				−31	−71	39
				35	36	38
				−45	32	31
				8	−47	30
				2	42	2
				44	−74	22
				−48	−71	24
				26	−68	45
				−43	−38	45
				38	34	30
				−40	40	24
Laird et al., 2009	Investigating the Functional Heterogeneity of the Default Mode Network Using Coordinate‐Based Meta‐Analytic Modeling.	840 (100%)	Talairach	−4	−58	44
				−4	−52	22
				2	32	−8
				52	−28	−24
				−2	50	18
				46	−66	16
				−26	16	44
				−56	−36	28
				−42	−66	18

Chiong et al., 2013	Moral reasoning task.	16 (control subjects only)	MNI	50	−6	26
				0	−78	42
				4	34	−14
				56	−64	26
				4	−58	24
				−20	4	−12
Poerio et al., 2017	Viewed a fixation cross with eyes open.	80 (100%)	MNI	−12	−92	−24
				−48	−72	−28
				−48	24	32
Spreng & Schacter, 2012	Visuospatial planning was investigated by presenting participants with 2 configurations on a single screen: the “goal” position and the “initial” position. The objective of this task was for the participant to determine the minimum number of moves to accomplish the goal state.	36 (100%)	MNI	−22	−22	−22
				47	−71	29
Yang et al., 2012	Subjects were asked to “relax and rest as we take pictures of your brain.”	18 (100%)	Talairach	−10	40	50
				10	66	10
				14	44	−8
				−64	−6	−8
				−60	−14	−20
				64	−8	−26
				−30	−22	−20
				36	−34	−18
Konishi et al., 2015	Participants alternated between task blocks in which they either make decisions about the location of shapes as they are presented on screen or with respect to their location on the prior trial.	29 (100%)	MNI	4	−64	30
				18	−52	16
				10	−46	0
				32	14	42
				22	42	40
				28	20	46
				0	36	22
				16	64	0
				28	56	8
				44	−58	36
				36	−62	26
				40	−68	44
				2	−32	36
				10	−46	34
				8	−16	30
				−24	−20	−18
				−22	−34	−8
				−16	−34	2
				−54	−8	−18
				−62	−18	−10
				−52	−16	−22
				−38	−72	36
				−48	−62	42
				−38	−74	28
				−8	−60	38
				−10	−38	28
				−2	−48	34
				−6	−66	6
				−18	−66	18
				−18	−64	8
Mason et al., 2007	Minds could wander when they performed practiced versus novel task sequences.	19 (100%)	Talairach	6	62	11
				0	62	16
				−12	49	45
				12	49	47
				21	40	45
				−38	20	48
				−9	−39	35
				6	−15	39
				24	−7	−20
				6	41	4
				−18	−36	−13
				−45	−68	37
				−9	−51	30
				42	−11	3
				45	−28	18
				−42	−18	−2
				−45	−63	31
				−45	−6	−7
				50	−57	33
				−30	−39	−13
				6	51	−9
				3	57	42
				3	−9	51
				−3	51	51
				−6	54	27
				−18	39	51
				15	48	42
				15	60	39
				21	−24	72
				6	48	9
				−6	−45	18
				−9	−42	27
				3	−45	18
				0	−9	39
				54	−57	36
				−48	−66	30
				6	−41	60
				45	−24	18
				−42	0	9
				30	−15	3
				36	−3	9
				−36	−9	9
				60	−6	3
				−45	−60	15
				−42	−37	3
Kennedy & Courchesne, 2008	Subjects made true/false judgments for various statements about themselves (SELF condition) or a close other person (OTHER condition).	13 (control only)	Talairach	−6	39	48
				−2	51	28
				2	−49	28
				−42	19	16
				−3	−97	24
				−42	3	48
				−50	−65	36
				−2	31	0
				−42	3	−28
				58	−65	28
				−50	−33	4
				55	−32	20
				7	39	−1
				26	−44	59
				51	−8	12
				6	−45	35
				10	−28	44
				−53	−61	24
				−37	−12	0
				−2	−89	20
				−6	−73	0
				22	−68	4
				7	−57	27
				−18	−45	4
				22	−45	7
				18	−25	17
				−38	28	0
				2	−16	32
				−1	51	24
				−2	20	20
				−5	−48	8
				−21	20	48
				3	−33	39
				−18	−33	−12
				−38	−73	29
				38	20	52
				47	−68	24
Xu et al., 2014	Nondirective meditation.	14 (100%)	MNI	8	37	10
				8	6	31
				−3	42	−3
Chen et al., 2017	Resting state	24 (control subjects only)	MNI	3	−54	27
				−45	−57	30
				−21	−33	−12
				−60	−9	−18
				−3	63	18

### 2.2 Creation of 3D Regions of Interest

In the original HCP study, parcellation data were analyzed using the CIFTI file format. CIFTI files use a surface‐based coordinate system termed greyordinates, which localizes regions of interest (ROIs) on inflated brains (Van Essen & Glasser, 2016). This is in contrast to traditional file formats, such as NIFTI, which denote regions based on volumetric dimensions (Larobina & Murino, 2014). As a result, it was difficult to perform deterministic tractography using ROIs in CIFTI file format. To convert the parcellations files to volumetric coordinates, the greyordinate label parcellation fields were standardized to the three‐dimensional volumetric working spaces of DSI Studio (Carnegie Mellon, http://dsi‐studio.labsolver.org) using the structural imaging data provided by the HCP. This operation was performed using Workbench Command within Connectome Workbench (Glasser et al., 2013). This allowed us to convert all 180 parcellations from surface‐based coordinates to volumetric coordinates and perform deterministic fiber tractography.

### 2.3 Activation likelihood generation and identification of relevant cortical regions

We used BrainMap Ginger ALE 2.3.6 to extract the relevant fMRI data for creation of an activation likelihood estimation (ALE; Eickhoff et al., 2009, 2012; Turkeltaub et al., 2012). All Talairach coordinates identified during literature review were converted to the MNI coordinate space using icbm2tal transform SPM Conversion in GingerALE. We subsequently performed a single study analysis using cluster‐level inference in the MNI coordinate space (cluster level of 0.05, threshold permutations of 1,000, uncorrected p‐value of 0.001). The ALE coordinate data were displayed on an MNI‐normalized template brain using the Multi‐image Analysis GUI (Mango) 4.0.1 (ric.uthscsa.edu/mango). The preconstructed ROIs of the HCP parcellations were overlaid on the ALE and two investigators independently compared the foci with the parcellations visually for inclusion in the DMN. Each investigator repeated the process to minimize intra‐observer variability. A third investigator reviewed the images and was available to resolve disputes in instances of interobserver variability, though this was seldom encountered.

### 2.4 Network tractography

Publicly available imaging data from the Human Connectome Project were obtained for this study from the HCP database (http://humanconnectome.org, release Q3). Diffusion imaging with corresponding T1‐weighted images from 25 healthy, unrelated subjects were analyzed during fiber tracking analysis (Subjects IDs: 100307, 103414, 105115, 110411, 111312, 113619, 115320, 117112, 118730, 118932, 100408, 115320, 116524, 118730, 123925, 148335, 148840, 151526, 160123, 178950, 188347, 192540, 212318, 366446, 756055). We used 25 brains as it is comparable to the number of subjects used in studies of a similar aim. We have previously tested the variability of tractography results above utilizing 25 subjects, and however, it was too small to justify using additional subjects as it is unlikely to alter the findings of the study. Often, beyond 10 subjects, the results do not change significantly. The demographics of the patients used in this study are detailed in Table 2. A multi‐shell diffusion scheme was used, and the b‐values were 990, 1985, and 1980 s/mm^2^. Each b‐value was sampled in 90 directions. The in‐plane resolution was 1.25 mm. The diffusion data were reconstructed using generalized q‐sampling imaging with a diffusion sampling length ratio of 1.25 (Yeh et al., 2010).

**TABLE 2:** Subject demographics

*Variable* (*n* = 25)	
Age (mean, *SD*, in years)	29.5 (3.8)
*Gender*	
Female (*n*, %)	13 (52)

All brains were registered to the Montreal Neurologic Institute (MNI) coordinate space (Evans et al., 1992), wherein imaging is warped to fit a standardized brain model comparison between subjects (Evans et al., 1992). Tractography was performed in DSI Studio (Carnegie Mellon, http://dsi‐studio.labsolver.org) using a region of interest approach to initiate fiber tracking from a user‐defined seed region (Martino et al., 2013). A two‐ROI‐approach was used to isolate tracts (Kamali et al., 2014).

Voxels within each ROI were automatically traced with a maximum angular threshold of 45 degrees. When a voxel was approached with no tract direction or a direction change of greater than 45 degrees, the tract was halted. Tractography was terminated after reaching a maximum length of 800 mm. In some instances, exclusion ROIs were placed to exclude obvious spurious tracts that were not involved in the white matter pathway of interest.

### 2.5 Measuring connection strength

To quantify the strength of the connections identified within the DMN across all subjects, the tracking parameters used within DSI Studio were modified such that the program would count the total number of tracts between any two ROIs based on a random seed count of 2.5 million. Working sequentially through ROI pairs in the network, the number of tracts between regions was recorded for each subject after fiber tractography was terminated under these new conditions. The connection strength between ROI pairs within the DMN was calculated by averaging the number of tracts between each ROI pair across all subjects.

## 3 RESULTS

### 3.1 ALE regions and their corresponding parcellations

**FIGURE 1:**
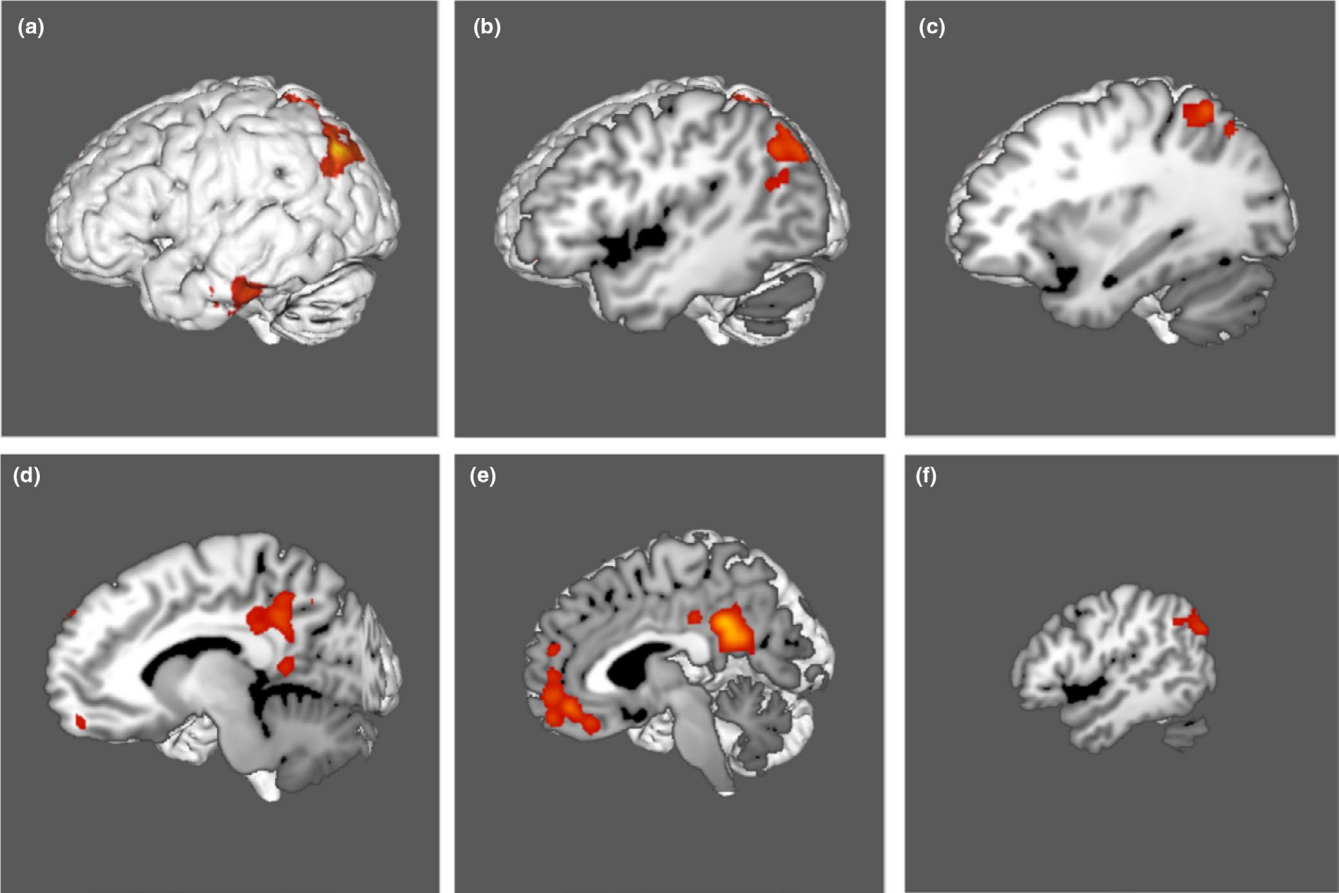
Activation likelihood estimation (ALE) of 27 resting‐state fMRI experiments related to goal‐oriented attentional processing. The three‐dimensional ALE data are displayed in Mango on a brain normalized to the MNI coordinate space. (a–c) ALE data highlighting the lateral parietal region. (d) ALE data highlighting the region of the posterior cingulate gyrus. (e) ALE data highlighting the anterior and posterior cingulate regions. (f) ALE data highlighting the lateral parietal region

**FIGURE 2:**
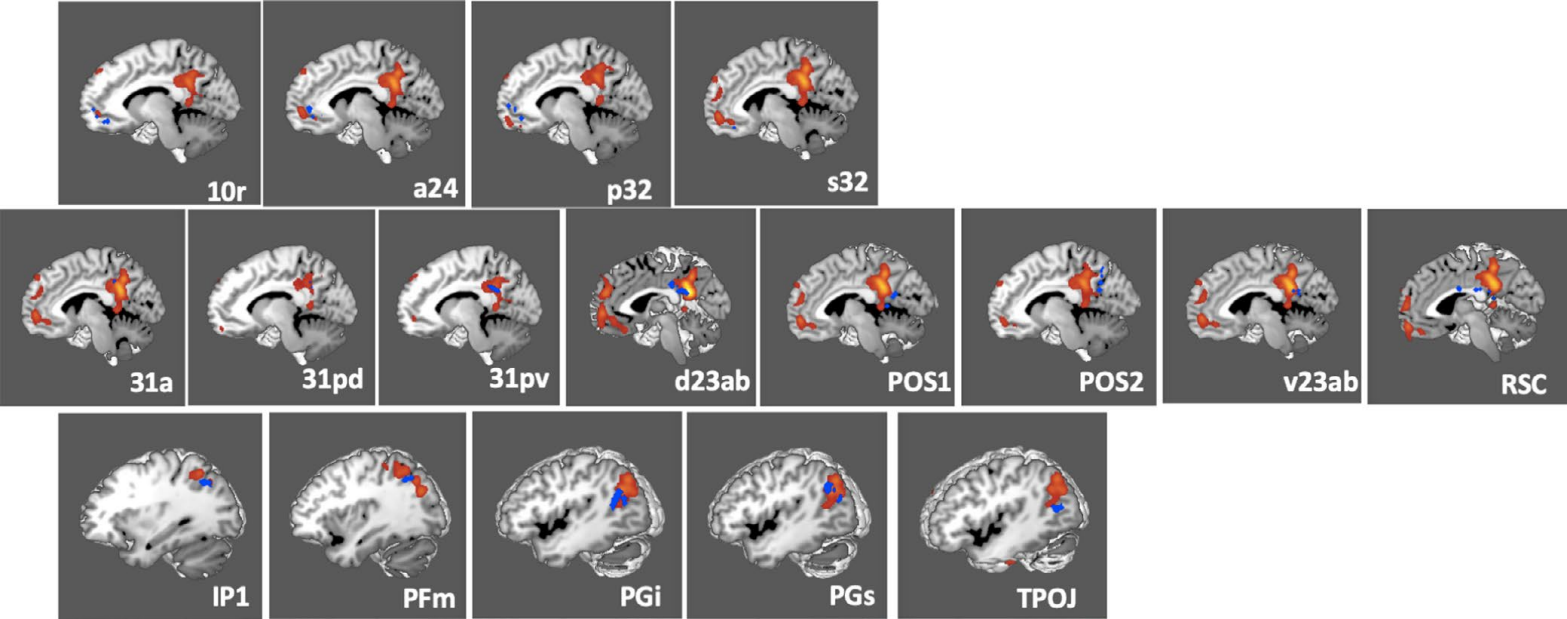
Comparison overlays between the cortical parcellation data (blue) and activation likelihood estimation (ALE) data (red) from Figure 1 in the left cerebral hemisphere. Regions were visually assessed for inclusion in the network if they overlapped with the ALE data. Parcellations included in the DMN model were identified in the anterior cingulate area including 10r, a24, p32, and s32 (top row); posterior cingulate area including 31a, 31pd, 31pv, d23ab, POS1, POS2, v23ab, and RSC (middle row); and lateral parietal area including IP1, PFm, PGi, PGs, and TPOJ3 (bottom row). The labels indicate the parcellation shown in each panel

Figure 1 demonstrates the ALE of the twenty‐seven MRI experiments included in our meta‐analysis. Highlighted areas include the bilateral posterior cingulate cortices, the anterior cingulate cortices, and the lateral parietal lobes. Seventeen regions of interest were found to overlap the fMRI data, including areas 10r, a24, p32, and s32 in the anterior cingulate cortex; areas 31a, 31pd, 31pv, d23ab, POS1, POS2, RSC, and v23ab in the posterior cingulate cortex and parieto‐occipital sulcus; and areas IP1, PFm, PGi, PGs, and TPOJ3 in the lateral parietal lobe. Comparison overlays between the cortical parcellations and the ALE data are shown in Figure 2.

### 3.2 Structural connections within the default mode network

**FIGURE 3:**
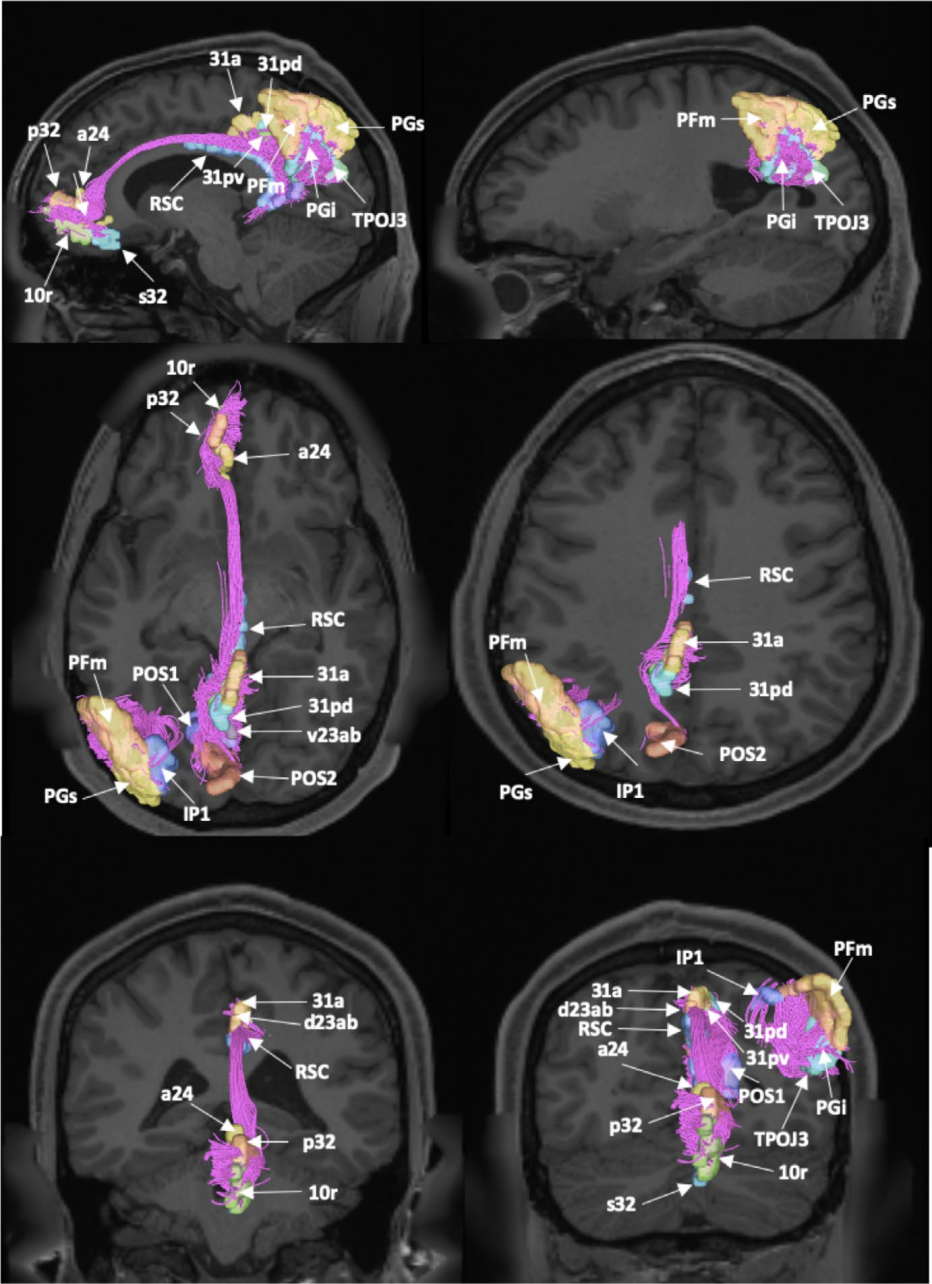
Fiber tracking analysis for the default mode network. Shown on T1‐weighted MR images in the left cerebral hemisphere. TOP ROW: sagittal sections from medial to lateral demonstrating the cingulum and its projections between the anterior and posterior cingulate clusters of the default mode network. MIDDLE ROW: axial sections from inferior to superior demonstrate the cingulum connecting the posterior cingulate and lateral parietal clusters. and the short fiber connections within the network. BOTTOM ROW: coronal sections from anterior to posterior provide another view of the cingulum and short fiber connections within the network

Deterministic tractography was utilized to show the basic structural connectivity of the DMN. These results are shown in Figure 3. Individual connections within the network are presented in Table 3 which tabulates the strengths of individual connections and lists the type‐specific white matter connections identified between regions.

**TABLE 3:** Type and strength of connections within the default mode network

Connection	*N*	Average strength weighted by all subjects	Average strength weighted by identified subjects	Connection type
*Anterior and Posterior Cingulate Connections*				
10r to 31pv	4	5.2	32.2	cingulum fiber
10r to a24	23	34.6	37.6	U‐shaped fiber
10r to d23ab	7	1.6	5.7	cingulum fiber
10r to p32	25	236.7	236.7	U‐shaped fiber
10r to POS1	13	18.4	35.4	cingulum fiber
10r to POS2	4	1.1	7.0	cingulum fiber
10r to RSC	14	13.0	23.2	cingulum fiber
10r to s32	21	128.7	153.2	U‐shaped fiber
10r to v23ab	10	13.2	32.9	cingulum fiber
31a to 31pd	25	177.4	177.4	U‐shaped fiber
31a to 31pv	24	133.9	139.5	U‐shaped fiber
31a to a24	5	3.7	18.4	cingulum fiber
31a to d23ab	24	61.0	63.5	U‐shaped fiber
31a to p32	6	2.0	8.3	cingulum fiber
31a to POS1	7	0.6	2.0	U‐shaped fiber
31a to POS2	14	18.7	33.4	U‐shaped fiber
31a to RSC	14	7.1	12.6	U‐shaped fiber
31pd to 31pv	25	203.6	203.6	U‐shaped fiber
31pd to a24	6	2.2	9.2	cingulum fiber
31pd to d23ab	13	7.6	14.7	U‐shaped fiber
31pd to p32	2	0.9	11.5	cingulum fiber
31pd to POS1	6	7.4	30.8	U‐shaped fiber
31pd to POS2	20	19.1	23.8	U‐shaped fiber
31pd to RSC	14	7.9	14.1	U‐shaped fiber
31pd to s32	1	0.04	1.0	cingulum fiber
31pd to v23ab	7	13.5	48.3	U‐shaped fiber
31pv to a24	16	10.7	16.7	cingulum fiber
31pv to d23ab	25	223.1	223.1	U‐shaped fiber
31pv to p32	11	18.8	42.8	cingulum fiber
31pv to POS1	20	34.9	43.6	U‐shaped fiber
31pv to POS2	20	25.4	31.8	U‐shaped fiber
31pv to RSC	23	28.1	30.5	U‐shaped fiber
31pv to s32	1	0.1	3.0	cingulum fiber
31pv to v23ab	25	120.6	120.6	U‐shaped fiber
a24 to d23ab	15	4.8	7.9	cingulum fiber
a24 to p32	23	191.6	208.3	U‐shaped fiber
a24 to POS1	16	25.4	39.6	cingulum fiber
a24 to POS2	5	2.4	12.2	cingulum fiber
a24 to RSC	22	42.5	48.3	cingulum fiber
a24 to s32	18	58.4	81.1	U‐shaped fiber
a24 to v23ab	15	8.6	14.3	cingulum fiber
d23ab to p32	14	7.2	12.8	cingulum fiber
d23ab to POS1	24	28.2	29.3	U‐shaped fiber
d23ab to POS2	15	29.4	49.0	U‐shaped fiber
d23ab to RSC	24	267.8	278.9	U‐shaped fiber
d23ab to v23ab	25	131.2	131.2	U‐shaped fiber
p32 to POS1	18	36.9	51.3	cingulum fiber
p32 to POS2	10	3.4	8.6	cingulum fiber
p32 to RSC	19	68.0	89.5	cingulum fiber
p32 to s32	20	43.4	54.3	U‐shaped fiber
p32 to v23ab	19	81.2	106.9	cingulum fiber
POS1 to POS2	25	174.4	174.4	U‐shaped fiber
POS1 to RSC	25	252.0	252.0	U‐shaped fiber
POS1 to s32	2	0.1	1.5	cingulum fiber
POS1 to v23ab	24	223.2	232.5	U‐shaped fiber
POS2 to RSC	17	15.1	22.2	U‐shaped fiber
POS1 to v23ab	11	2.3	5.3	U‐shaped fiber
RSC to s32	2	1.8	23	cingulum fiber
RSC v23ab	24	136.4	142.1	U‐shaped fiber
s32 to v23ab	2	0.2	2.0	cingulum fiber
*Lateral Parietal Connections*				
IP1 to PFm	23	149.4	162.4	U‐shaped fiber
IP1 to PGi	12	19.9	41.4	U‐shaped fiber
IP1 to PGs	20	145.6	182.0	U‐shaped fiber
IP1 to TPOJ3	7	1.0	3.7	U‐shaped fiber
PFm to PGi	25	418.5	418.5	U‐shaped fiber
PFm to PGs	17	142.8	210.0	U‐shaped fiber
PFm to TPOJ3	5	1.0	4.8	U‐shaped fiber
PGi to PGs	24	220.3	229.5	U‐shaped fiber
PGi to TPOJ3	25	211.2	211.2	U‐shaped fiber
PGs to TPOJ3	18	39.8	55.3	U‐shaped fiber

The cortical areas identified as part of the DMN can be grouped into three distinct clusters: an anterior cingulate cluster (10r, a24, p32, s32), a posterior cingulate cluster (31a, 31pd, 31pv, d23ab, POS1, POS2, RSC, v23ab), and a lateral parietal cluster (IP1, PFm, PGi, PGs, TPOJ3). U‐shaped fibers form a majority of the connections between ROI pairs of the network. These fibers generally have the same morphology, arising within one part of the cortex before curving 180 degrees to terminate in a part of the brain immediately adjacent to its origin. These U‐shaped fibers represent the local connections between anterior cingulate, posterior cingulate, and lateral parietal areas in close proximity.

The cingulum was also identified during fiber tracking analysis. This white matter bundle was found to connect the anterior and posterior cingulate clusters within the DMN. These fibers arise from the anterior cingulate cortex, and curve posteriorly to run within the deep white matter adjacent to the cingulate gyrus. The fibers course along the length of the corpus callosum, until they terminate in the posterior cingulate cortex and parieto‐occipital sulcus (Figure 3).

All four parcellations of the anterior cingulate cluster (10r, a24, p32, and s32) contribute to the cingulum, though with variable frequency. Areas a24 and p32 demonstrated consistent connections across all 25 subjects to all parcellations of the posterior cingulate cluster (31a, 31pd, 31pv, d23ab, POS1, POS2, RSC, and v23ab). In contrast, the connections from areas 10r and s32 were occurred infrequently, and the parcellations were found to connect to fewer regions of the posterior cingulate cortex (Table 3).

**FIGURE 4:**
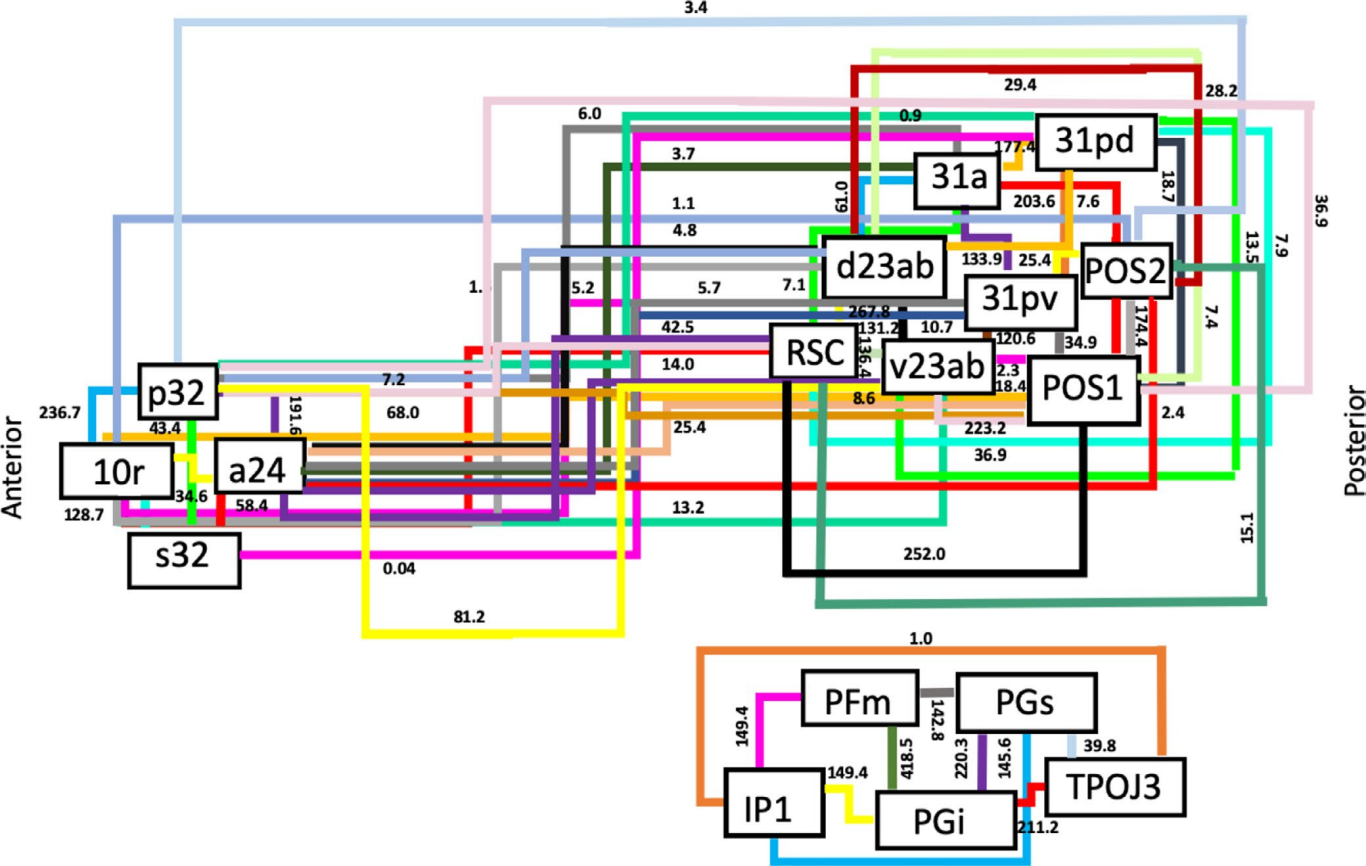
Simplified schematic of the white matter connections identified between individual parcellations of the default mode network during fiber tracking analysis. Connections are labeled with the average strength measured across all 25 subjects

No long‐association fiber bundle was found to connect the lateral parietal regions to either the anterior cingulate or posterior cingulate cortices. This was expected, as no such connection has been described previously. However, IP1, PFm, PGi, PGs, and TPOJ3 all showed consistent interconnections between one another in the form of U‐shaped fibers. Figure 4 is a simplified schematic of the connections discussed above. Lines in this schematic represent individual connections of the DMN and are labeled with their corresponding strength measured by averaging the number of tracts between ROI pairs across all subjects.

There was no relationship between demographic data and network anatomy within our cohort. Our cohort was not diverse enough to observe significant differences as this was not a primary aim of this study and we wanted to produce a model from healthy controls to avoid confounding factors. It may, however, be interesting to study changes in the DMN under different demographic characteristics in the future.

## 4 DISCUSSION

In this study, we utilized meta‐analytic software and deterministic tractography to construct an anatomic model of the DMN based on the cortical parcellation scheme previously published under the Human Connectome Project (Glasser et al., 2016). The DMN is a critical resting‐state brain network involved in mind‐wandering, and mental time travel, though it is also active during moral reasoning, autobiographical and episodic memory retrieval, and semantic processing (Alves et al., 2019; Buckner et al., 2008; Horn et al., 2014; Murphy et al., 2018). It is also implicated in several disorders including depression, autism, and dementia (Dutta et al., 2019; Ouchi & Kikuchi, 2012; Padmanabhan et al., 2017). Accurately defining regions of the DMN are pivotal to understanding its function which may then offer insights for clinicians into the mechanism and potential therapies for these disorders. In addition, a precise anatomic and connectomic description of the network will allow surgeons to make better judgments during brain surgery. The anatomic constituents of this network are discussed below.

### 4.1 The anterior cingulate cluster

Cortical areas 10r, a24, p32, and s32 overlap with the ALE in the region of the anterior cingulate cortex, which has been identified as a component of the DMN in multiple studies (Andrews‐Hanna et al., 2014; Buckner et al., 2008; Mars et al., 2012; Schilbach et al., 2008). Area 10r is one of several newly described divisions of the original Brodmann Area 10 (Glasser et al., 2016), which expanded the entire frontal polar cortex from the medial superior frontal gyrus to the dorsolateral prefrontal cortex (Burgess & Wu, 2013; Peng et al., 2018). Little is known about this region; however, it is located in the anterior inferior portion of the medial superior frontal gyrus. Just posterior to area 10r is area s32 which lies in the subcallosal cortex. This region is interconnected to other areas of the limbic system and is known to play a role in emotional response regulation and reward expectation (Beckmann et al., 2009; Palomero‐Gallagher et al., 2008).

Superior to areas 10r and s32 are areas p32 and a24, respectively. Area p32 is located in the antero‐medial superior frontal gyrus, bordering the inferior bend of the callosal sulcus. This region plays a role in the integration of emotional and cognitive information during social interaction tasks to assist in error monitoring (Beckmann et al., 2009; Palomero‐Gallagher et al., 2008). Area a24 is located in the anterior cingulate gyrus proper, lying anterior to the genu of the corpus callosum. This region has been implicated as part of the “affect division” of the anterior cingulate cortex and has been linked to the analysis of internal and external states of mind to assist in emotional expression and motivation (Devinsky et al., 1995; Drevets et al., 2008).

All four areas are interconnected by U‐shaped fibers to one other and demonstrate fiber projections consistent with the cingulum to the posterior cingulate cortex.

### 4.2 The posterior cingulate cluster

Cortical areas 31a, 31pd, 31pv, d23ab, POS1, POS2, RSC, and v23ab overlap with the ALE in the region of the posterior cingulate cortex, which has also been identified consistently as a component of the DMN across multiple studies (Che et al., 2014; Leech & Sharp, 2014; Lin et al., 2017; Luo et al., 2016; Wang et al., 2019). Based on their functionality and anatomic distribution, these regions can be further classified into dorsal and ventral posterior cingulate regions.

Areas 31a and d23ab comprise the dorsal division of parcellations of the posterior cingulate cortex within the DMN. Area 31a is located on the anterior half of the subparietal gyrus, directly posterior to the marginal sulcus. Area d23ab is located on the posterior cingulate gyrus proper, superior to the splenium of the corpus callosum. Both areas are considered part of the dorsal posterior cingulate cortex which is highly active during tasks that require an external focus, especially concerning visuospatial orientation of the body (Aggleton et al., 2014; Bzdok et al., 2015; Glasser et al., 2016; Leech & Sharp, 2014). Task functional magnetic resonance imaging also indicates that these regions are involved in the working memory processes related to places and body images (Aggleton et al., 2014; Bzdok et al., 2015; Glasser et al., 2016; Leech & Sharp, 2014).

In contrast to areas 31a and d23ab, areas 31pd, 31pv, and v23ab comprise the ventral division of parcellations of the posterior cingulate cortex within the DMN. Area 31pd is located on the posterior superior portion of the subparietal gyrus, while area 31pv is located on the posterior inferior portion of the subparietal gyrus where it extends across the cingulate sulcus onto the posterior cingulate gyrus. Area v23ab is located in along the caudal aspect of the posterior cingulate cortex, near the superior portion of the cingulate isthmus. These areas are considered part of the ventral posterior cingulate cortex which is active during self‐relevant tasks, including retrieval of semantic and episodic memories (Aggleton et al., 2014; Bzdok et al., 2015; Glasser et al., 2016; Leech & Sharp, 2014). Task fMRI studies indicate that these regions are also involved in the working memory processes of body and face images (Aggleton et al., 2014; Bzdok et al., 2015; Glasser et al., 2016; Leech & Sharp, 2014).

Three other areas were identified as forming part of the posterior cingulate cluster within our anatomic model of the DMN: RSC, POS1, and POS2. These regions do not sort easily into either the ventral or dorsal divisions of the DMN. The RSC, or retrosplenial cortex, is a thin region of the cortex that occupies the inferior aspect of posterior cingulate cortex. It lies immediately adjacent to the callosal sulcus and wraps around the splenium of the corpus callosum. This region is primarily responsible for transitioning between allocentric or view‐independent spatial perspectives and egocentric or view‐dependent spatial perspectives (Aggleton et al., 2014; Glasser et al., 2016; Leech & Sharp, 2014; Vann et al., 2009). The RSC is implicated in spatial navigation, episodic memory, future planning, and imagination. In addition, the RSC has been suspected of being involved in the retrieval of recent autobiographical information from memory (Aggleton et al., 2014; Glasser et al., 2016; Leech & Sharp, 2014; Vann et al., 2009).

Regions POS1 and POS2 occupy the inferior and superior halves of the anterior bank of the parieto‐occipital sulcus, respectively. Task fMRI studies demonstrate that areas POS1 and POS2 are activated during the working memory processes of place images (Glasser et al., 2016), and it has been suggested that both regions have a strong, coupled functional correlation with area RSC related to scene comprehension (Glasser et al., 2016).

All eight regions are interconnected by U‐shaped fibers to one other and demonstrate fiber projections consistent with the cingulum to the anterior cingulate cortex. Other studies have reported that a component of the superior longitudinal fasciculus, SLF‐I, connects regions of the precuneus to more anterior regions within the frontal lobe. We were unable to identify fibers of the superior longitudinal fasciculus on tractography. We suspect this may have been due to the SLF‐I’s greater involvement in connecting the precuneus to more dorsal regions of the frontal lobe. There are proposed fibers of the SLF‐I which run closely parallel to the cingulum, namely the paracingulate fascicle, though the validity of performing such segregation on DTI, and whether this segregation has purposeful meaning on function is not clear.

### 4.3 The lateral parietal cluster

Cortical areas PFm, PGi, PGs, IP1, and TPOJ3 overlap with the ALE in the region of the lateral parietal lobe, which, as for the cingulate cortices, has been identified consistently as a component of the DMN across multiple studies (Alves et al., 2019; Buckner et al., 2008; Lei et al., 2014; Sestieri et al., 2011; Xu et al., 2016; Zanchi et al., 2017). Many of these parcellations are located in the inferior parietal lobule, including areas PFm, PGs, and PGi.

Area PFm is located on the anterior superior surface of the angular gyrus and extends across the sulcus onto the posterior superior bank of the supramarginal gyrus. In contrast, areas PGs and PGi are contained entirely within the angular gyrus, occupying its superior and inferior surfaces, respectively. The functions attributed to these regions are numerous and are summarized below:

‐ Area PFm has been shown to be active during nonspatial attention tasks, during decision making tasks when individuals change choices, during rule changes during visually guided attention tasks, and during attentional reorientation (Caspers et al., 2006). The inferior parietal lobule is also involved in the syntactical components of language processing (Ben Shalom & Poeppel, 2008).
‐ Area PGs has been shown to be active when individuals change their visuospatial attention from one focus to another (Mars et al., 2011). Specifically, area PGs is involved in the response to biological motion (Mars et al., 2011). The region is also relevant in number processing (Caspers et al., 2011).
‐ Area PGi has a functional profile similar to that of area PGs. For example, area PGi has been shown to be active when individuals change their visuospatial attention from one focus to another (Mars et al., 2011). Within the original parcellations study, the Human Connectome Project authors discuss both areas PGi and PGs as major nodes in the DMN (Glasser et al., 2016).

The other two areas identified as part of the DMN were areas IP1 found on the inferior bank of the intraparietal sulcus, and area TPOJ3 found on the posterior inferior portion of the inferior parietal lobule. Area IP1 shows significant activation during mental arithmetic activities, and, as part the intraparietal sulcus, supports more complex parts of numeric and mathematical information processing (Uddin et al., 2010; Wu et al., 2009). The functional profile of area TPOJ3 is not as well characterized.

The three clusters of the DMN we identified in our study aligns with other studies defining the DMN. Our aim was indeed to define the DMN using standardized and precise anatomical nomenclature. Recently, however, one study extended the definition of the DMN to include the thalamus and basal forebrain through aligning structural MRI data with functional maps (Alves et al., 2019). Moreover, we were also unable to identify previously defined components of the DMN in the temporal lobe, including the parahippocampal region and middle temporal gyrus. We suspect this may have been due to our inclusion criteria relying on the complete reporting of MNI coordinates by original studies, which was not uniformly done. Nevertheless, these regions, combined with the basal forebrain and thalamus, may explain the role of the DMN in memory, as reported by previous studies showing DMN activation during memory tasks (Huo et al., 2018; Spreng & Grady, 2010). This may consequently provide new avenues for exploring pathogenesis and treatment in pathological states such as Alzheimer's disease, where a decreased functional connectivity in the DMN has been observed (Qi et al., 2018).

### 4.4 The strength of connections within the default mode network

The strength of the connections identified between parcellations of the DMN is reported in Table 3. Two different values for strength are recorded based on the average number of tracts across all subjects versus the average number of tracts across subjects in which the connection was actually identified. It is certainly the case that the structural connectivity of the DMN varies to some degree between individuals, and by presenting both sets of average connectional strengths, one can see how connections can vary in the network. For example, the cingulum projection from area 10r to area 31pv has an average strength of 5.2 across all 25 subjects (meaning one would expect to find 5.2 streamlines using the fiber tracking algorithm discussed in the methods) versus an average strength of 32.2 in the four individuals in which the connection was identified. By reporting both numbers, we can see that, while the connection between 10r and 31pv occurs infrequently in the network, in individuals who have such a connection, it is relatively strong.

It should also be noted that we did not set a threshold for the strength that might limit the connections shown for the DMN. For example, assessing the connection between a24 and d23ab via the cingulum, one sees that the average strength across all 25 subjects used in this study was 4.8 versus 7.9 in the fifteen subjects for whom such a connection was actually identified. If we had set a threshold of an average strength of 10.0 or set a threshold related to the frequency by which we saw the connection, that is, in at least 20/25 subjects, then we would not report this connection at all. In our mind, this is incorrect. Instead, it more appropriate to say that the connection between a24 and d23ab, while relatively weak compared to other connections in the network, occurs relatively frequently within the DMN. This is as opposed to reporting that no such connection exists between the two parcellations.

Despite not setting a threshold for network connectivity, the frequency and strength associated with certain connections identified as part of the DMN (e.g., the connection between 31pd and s32 which was identified in one subject with an overall strength of 0.04) raise an important question of which connections are critical for the functionality of the network. Answering this question is beyond the scope of this study, and further research is needed to understand which connections within the DMN are most critical for the successful functioning of the DMN.

## 5 CONCLUSIONS

We present a preliminary anatomic model of the default mode network. Further studies may refine this model with the ultimate goal of clinical application.

## CONFLICT OF INTEREST

Dr. Sughrue is the Chief Medical Officer of Omniscient Neurotechnologies. No products directly related to this were discussed in this paper. All other authors have no financial interest or potential conflicts of interest.

## ETHICAL STATEMENT

The current study does not have any ethical considerations as it is a meta‐analysis on previously completed data.

### Peer Review

The peer review history for this article is available at https://publons.com/publon/10.1002/brb3.1976.

## Data Availability

The data that support the findings of this study are available from the corresponding author (MS), upon reasonable request.
